# The interplay between cell wall integrity and cell cycle progression in plants

**DOI:** 10.1007/s11103-023-01394-w

**Published:** 2023-12-13

**Authors:** Nancy Soni, Laura Bacete

**Affiliations:** 1 Faculty of Natural Sciences, Institute for Biology, Norwegian University of Science and Technology, 5 Høgskoleringen, 7491 Trondheim, Norway; 2 Department of Plant Physiology, Umeå Plant Science Centre (UPSC), Umeå University, 901 87 Umeå, Sweden

**Keywords:** Plant cell wall integrity, Cell cycle progression, Endoreplication, Auxin, Cytokinin, Cell wall sensing, Growth-defense trade-off

## Abstract

Plant cell walls are dynamic structures that play crucial roles in growth, development, and stress responses. Despite our growing understanding of cell wall biology, the connections between cell wall integrity (CWI) and cell cycle progression in plants remain poorly understood. This review aims to explore the intricate relationship between CWI and cell cycle progression in plants, drawing insights from studies in yeast and mammals. We provide an overview of the plant cell cycle, highlight the role of endoreplication in cell wall composition, and discuss recent findings on the molecular mechanisms linking CWI perception to cell wall biosynthesis and gene expression regulation. Furthermore, we address future perspectives and unanswered questions in the field, such as the identification of specific CWI sensing mechanisms and the role of CWI maintenance in the growth-defense trade-off. Elucidating these connections could have significant implications for crop improvement and sustainable agriculture.

## Key message

In this review, we provide a comprehensive exploration of the intricate relationship between plant CWI and cell cycle progression. By drawing parallels from studies in yeast and mammals, we offer new insights into this critical facet of plant cell biology. We underscore the role of endoreplication in cell wall composition and the molecular mechanisms linking CWI perception to cell wall biosynthesis and gene expression regulation. The review also identifies key research avenues that remain to be explored, such as specific CWI sensing mechanisms and the role of CWI maintenance in the growth-defense trade-off. Elucidating these links holds significant potential for crop improvement and sustainable agriculture, thus rendering our work of broad interest to researchers in plant biology, agricultural science, and sustainable farming practices.

## Introduction

In contrast to animal cells, plant cells have a sturdy and organized protective extracellular matrix known as the plant cell wall. Plant cell walls are dynamic, complex structures rich in polysaccharides (cellulose, hemicelluloses, and pectins), playing a critical role in plant growth and development. Given the crucial functions of the plant cell wall, plants must possess the ability to perceive and maintain its structural integrity. This enables them to initiate restorative processes when needed. The maintenance of cell wall integrity (CWI) in plants involves specialized mechanisms initially identified in yeast (Bacete and Hamann 2020). These CWI mechanisms allow plants to respond adaptively to changes in cell walls created by both internal and external stimuli, which is essential for plant plasticity. Hence, CWI mechanisms consistently survey cell wall functional integrity, initiating compensatory changes in cell walls and metabolism to maintain integrity in the face of developmental processes and stress conditions. In the model plant *Arabidopsis thaliana*, the CWI maintenance mechanism uses both osmo- and mechano-perception to detect and respond to cell wall damage (Gigli-Bisceglia et al. 2018; Bacete and Hamann 2020; Bacete et al. 2022). Notwithstanding its importance, how changes in CWI impact cell cycle progression remains unclear, highlighting the need for further research in this area.

This review aims to investigate the intricate interconnections between the cell wall and cell cycle in plants, particularly how CWI regulates cell cycle progression. By drawing on insights from yeast and mammals, we will explore the lesser-known regulatory pathways controlling cell cycle activity in plants in response to changes in CWI. Further understanding of these connections has potential implications for crop improvement and the advancement of sustainable agriculture.

## Overview of the cell cycle

**Fig. 1:**
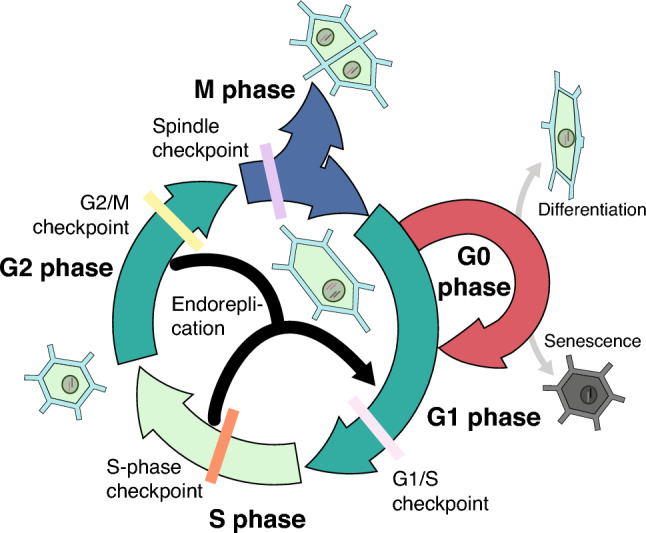
Diagram of the cell cycle. The key stages of the plant cell cycle, Synthesis (S) and Mitosis (M) phases, are separated by gap phases (G1 and G2). Regulatory checkpoints at G1/S, G2/M transitions, during S phase, and before anaphase, are crucial for maintaining DNA fidelity and regulating cell cycle progression. The term G0 is used in plants for meristematic quiescence or terminal differentiation, but its definition is unclear. In addition to the regular cell cycle, plants also exhibit a cell cycle variant known as endoreplication, in which nuclear DNA is replicated without subsequent cell division, resulting in increased ploidy levels or polyploidy

The cell cycle, a central process in all living organisms, enables cells to grow, replicate their genetic material, and then segregate the copies into two genetically identical daughter cells (Fig. 1). Its core components and progression mechanisms are shared across the biological spectrum. Yet, there are unique variations worthy of exploration. The interplay between the cell cycle and the extracellular matrix represents a fascinating area of study, particularly in organisms characterized by strong cell walls like yeast and plants. This section aims to present a comprehensive understanding of the cell cycle, its regulatory processes, and the unique characteristics of the plant cell cycle.

### Cell cycle and its phases

Cell division is a cornerstone process of cell biology, encompassing mitosis and meiosis in eukaryotic cells. This section will concentrate primarily on mitosis, a process that allows each new cell to receive a full set of chromosomes, thus conserving genetic consistency. Unlike prokaryotic cells that divide by a simpler method called binary fission, eukaryotic cells undergo a more complex division process. The process defines a series of events sequentially to ensure the proper chromosome duplication and segregation. The eukaryotic cell cycle is typically divided into four main phases: G1 (Gap 1), S (Synthesis), G2 (Gap 2), and M (Mitosis) (Fig. 1). The G0 phase is a period of quiescence or differentiation where cells are not actively dividing (Pardee 1974) (Fig. 1). While well-defined in animals, its definition in plants is unclear due to varied interpretations and limited molecular distinctions from a prolonged G1 state. Despite this ambiguity, G0 cells can re-enter the cell cycle under favorable conditions, except for differentiated and/or senescent cells (Velappan et al. 2017).

G1, S, and G2 together are called interphase, which occupies about 23 h of a 24-h cycle in a typical human cell proliferating in culture. The remaining hour is taken up by mitosis, during which the cell’s chromosomes are divided and two new daughter cells are formed (Alberts et al. 2002). The cell cycle duration in Arabidopsis and yeast is significantly shorter than in human cells. In Arabidopsis, the cell cycle duration in the root meristem is typically around 15–20 h (Beemster et al. 2005), while in yeast, the cell cycle duration is typically around 90 min (Hartwell et al. 1974). However, the cell cycle duration for pericycle cells engaged in lateral root initiation in Arabidopsis has been reported to be as short as 8 h. This suggests that the cell cycle can be accelerated during lateral root development, which is important for the rapid formation of new lateral roots (Alberts et al. 2002).

The two major cell cycle phases are DNA duplication during the S phase, which takes around 10–12 h for the proper duplication: and chromosome segregation during the M phase, which takes less than an hour. M phase includes mitosis, where the duplicated chromosomes condense, the nuclear envelope breaks down, and the chromosomes align at the equator of the mitotic spindle during metaphase. This is followed by anaphase, where sister chromatids separate and move to opposite spindle poles. Finally, cytokinesis results in the complete division of the cell. Compared to replicating DNA and division, most cells require much more time to grow and double their mass of proteins and organelles. This leads to the insertion of extra gap phases in most cell cycles to allow more time for growth. A G1 phase between the M phase and S phase is inserted where the primary focus of the cells is on growth and metabolic activities. A G2 phase between the S and mitosis allows cells to grow further and prepare for mitosis (Alberts et al. 2002).

### Mechanisms controlling cell cycle progression

Checkpoints and checkpoint pathways are crucial in overseeing the cell cycle’s integrity, ensuring DNA integrity before replication and segregation, and preventing genetic errors. There are four primary checkpoints (Hartwell and Weinert 1989): the G1–S checkpoint, where the cell assesses DNA integrity before entering the S phase; the S checkpoint, monitoring DNA synthesis to ensure accurate replication; the G2–M checkpoint, verifying that DNA replication is complete and undamaged before entering mitosis; and the spindle checkpoint, which ensures proper attachment of chromosomes to the spindle fibres before allowing the cell to progress from metaphase to anaphase during cell division. These checkpoints (Fig. 1) contribute to the precise regulation of the cell cycle, safeguarding against errors that could lead to genomic instability and cellular dysfunction.

The cell cycle is meticulously regulated at these checkpoints, primarily driven by the intricate interplay between cyclin-dependent kinases (CDKs) and cyclins. Cyclins are regulatory proteins that determine the progression of the cell cycle by activating CDKs. In plants, cyclins such as A-, B-, and D-type cyclins show distinct roles in the cell cycle, dictating the timing of cell cycle transitions (Inzé and De Veylder 2006).

CDKs, a highly conserved group of serine/threonine kinases, form complexes with specific cyclins at different cell cycle stages, thereby facilitating the phosphorylation of key target proteins necessary for advancing the cell cycle. Arabidopsis genome codes for about 30 CDKs and CDK-like proteins, illustrating the complexity of CDK regulation in plants (Menges et al. 2005). In the context of plant cell cycle regulation, CDKs are organized into eight groups, with CDKA and CDKB playing central roles (Vandepoele et al. 2002). CDKA’s activity peaks at G1/S and G2/M transitions (Inzé and De Veylder 2006; Gutierrez 2009), while CDKBs exhibit distinct expression patterns during the cell cycle (Inzé and De Veylder 2006).

CDK inhibitors (CKIs) are vital in cell cycle regulation across eukaryotes. In yeast, CKIs like Sic1 control the G1 phase, preventing premature S phase entry (Schwob 1994). In animals, two CKI families, INK4 and Cip/Kip, target specific CDKs in response to various cellular signals (Sherr and Roberts 1999). In contrast, plant CKIs such as ICK1/KPR1 and ICK2/KRP2in Arabidopsis affect both mitotic and endoreduplication cycles, crucial for plant development and environmental adaptation (Wang et al. 2006). This regulatory complexity in plants extends to other key proteins that have adapted distinct functions in plants different from their well-defined roles in yeast and animals. For example, the WEE1 kinase in humans, yeast, and plants inhibits cell division by phosphorylating CDKs, but its roles differ significantly beyond this (Détain et al. 2021). In plants, WEE1 is crucial in stress responses, especially to DNA damage and environmental stresses like drought and salinity (Harashima et al. 2013; Crncec and Hochegger 2019). Additionally, the importance of WEE1 in development varies among plant species; for example, it’s critical in tomato development but not in Arabidopsis (De Schutter et al. 2007; Gonzalez et al. 2007). This highlights how plants have uniquely adapted familiar cell cycle components to suit their specific life processes.

Transcriptional regulation is also essential for cell cycle regulation, and thus transcription factors have a prominent role. For example, in humans, thousands of enhancer RNAs and associated transcription factors exhibit a strong association with the transcription regulated by the cell cycle (Liu et al. 2017). In the plant kingdom, a good example is the transition into the M phase, where the orchestration involves the interplay of G2/M-specific genes and their promoter-bound mitosis-specific activator (MSA) element. This process is regulated by R1R2R3-type MYB transcription factors (Chen et al. 2017). The MYB3R family comprises both activators (Act-MYB) and repressors (Rep-MYB), and their intricate interplay governs the surge in mitotic CDK activity before entering M phase (Chen et al. 2017). Moreover, MYB3Rs interact with RBR protein and E2Fs, forming a large protein complex named the DREAM/dREAM-like complex, involved in regulating proliferative and quiescent states (Magyar et al. 2016; Umeda et al. 2019). These factors coordinate various cell cycle regulators, ensuring cells enter mitosis only when prepared.

### Specific features of the plant cell cycle

#### Cytokinesis and phragmoplast formation

Cytokinesis is a fundamental process in plant development that divides the cytoplasm of a dividing cell into two daughter cells. It is fundamentally different from cytokinesis in animals and fungi, and involves the de novo formation of a cell plate (Sinclair et al. 2022). The process starts with the phragmoplast guiding cytokinetic vesicles to the cell division plane. Here, vesicles fuse to form a cell plate, with callose deposition playing a key role in its stiffening and maturation (Otegui et al. 2001; Seguié-Simarro et al. 2004; McMichael and Bednarek 2013; Smertenko et al. 2018). This intricate process is orchestrated by a complex interplay of molecular components, including microtubules, microfilaments, and associated proteins like myosins and kinesins. In Arabidopsis, the preprophase band (PPB) and TON1/TRM/PP2A complex determine the division plane early in mitosis (Van Damme et al. 2007; Spinner et al. 2013). To ensure successful expansion and maturation of the phragmoplast, proteins like myosin VIII, myosin XI members, Kinesin-12 POK1 and POK2, TAN1, AIR9, PHGAPs, and IQ67 DOMAIN (IQD) proteins contribute to its structural integrity (Wu and Bezanilla 2014; Stöckle et al. 2016; Abu-Abied et al. 2018; Müller 2019). Proteins like KATANIN1 and MACET4/CORD4 are vital for phragmoplast organization, while SNARE proteins such as KNOLLE and SNAP33 drive vesicle fusion and membrane organization (Lauber et al. 1997; Zhang et al. 2011; El Kasmi et al. 2013; Jürgens et al. 2015; Karnahl et al. 2017; Sasaki et al. 2019; Panteris et al. 2021). Small GTPases like RABA2 and RABA3 ensure precise vesicle targeting, and complexes like TRAPPII and the exocyst regulate cell plate assembly (Chow et al. 2008; Berson et al. 2014; Rybak et al. 2014). Membrane recycling through clathrin-coated vesicles, involving proteins like Clathrin Light Chain and Dynamin-Related Proteins, also plays a crucial role (Fujimoto et al. 2008; McMichael and Bednarek 2013).

In Arabidopsis, small GTPases, including RABA2, RABA3, and RABA1 members, ensure vesicle targeting (Chow et al. 2008; Berson et al. 2014), while tethering complexes like TRAPPII and exocyst sequentially regulate cell plate assembly, expansion and maturation (Rybak et al. 2014). Moreover, membrane recycling via clathrin-coated vesicles plays a pivotal role during cytokinesis, with a range of associated proteins involved, including Clathrin Light Chain, Dynamin-Related Proteins, SCD1 and 2, Epsin-like adaptors, and the T-PLATE. These intricate molecular processes ensure the successful formation of the cell plate during plant cytokinesis, a fundamental step in cell division and plant growth.

Recent studies have highlighted the species-specific nature of cytokinesis. Research on the impact of cytokinesis inhibitors like Endosidin7 (ES7) and microtubule disruptors such as chlorpropham (CIPC) demonstrates the complex and varied responses in plant cytokinesis in Arabidopsis and maize (Allsman et al. 2023). ES7 induced cell plate defects in Arabidopsis without affecting callose accumulation or cell plate formation in maize. In contrast, CIPC treatment in maize occasionally led to irregular cell plates that split or fragmented but left cell-plate protein accumulation intact. This underlines the multifaceted regulation and the adaptive aspects of this crucial cellular process in different plant species.

#### Endoreplication and its role in plant development

Endoreplication, a cellular process also known as the endocycle, is a common process in plants. It involves multiple rounds of DNA synthesis without cell division, resulting in polyploid cells with increased DNA content. This unique mechanism allows for enhanced cellular functions such as vibrant colours, improved nutrient storage, and stress resistance (Edgar and Orr-Weaver 2001; Orr-Weaver 2015). It is especially prominent in higher plants and significant in certain cell types like the endosperm, contributing to metabolic activity, cell differentiation and rapid cell growth (Edgar and Orr-Weaver 2001; Bhosale et al. 2019).

The process of endoreplication involves multiple G and S phases leading to increased genetic material (Fig. 1) (De Veylder et al. 2002; Cook et al. 2013). This process is intricately regulated by a balance between CDK-cyclin complexes and CDK inhibitors, such as the SIAMESE/SIAMESE RELATED (SIM/SMR) family, and involves the degradation of cyclins by the anaphase-promoting complex/cyclosome (APC/C) (De Veylder et al. 2002, 2011; Cook et al. 2013). These pathways are modulated by protein complexes, phytohormones and biostimulators (Kołodziejczyk et al. 2021). In particular, auxin significantly influences the switch from mitotic cycles to endocycles. High levels of auxin signaling maintain cells in mitotic cycles possibly through the expression of CYCLIN A2;3 (CYCA2;3), while lower levels prompt a transition to endocycles (Ishida et al. 2010). Transcription factors also play a role, with factors like MED16, LMI1, SOG1, and E2Fa influencing the switch between mitotic cycles and endoreplication (Kołodziejczyk et al. 2021).

## Extracellular matrix and cell cycle regulation: what do we know from other organisms?

**Fig. 2:**
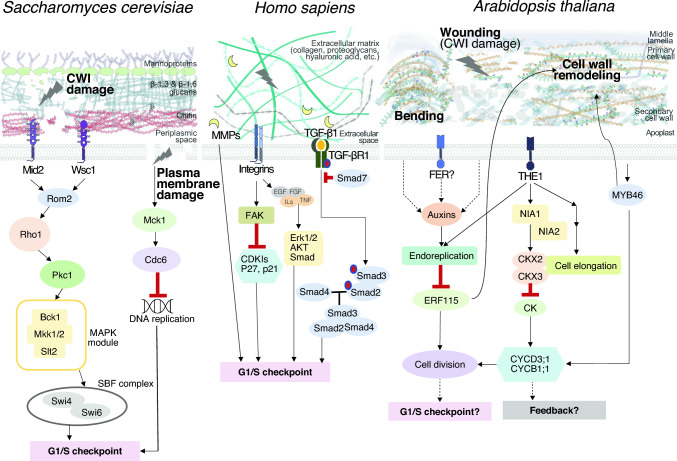
Pathways controlling cell cycle progression in function of the integrity of the cell wall or extracellular matrix are similar in different eukaryotes. In the yeast *Saccharomyces cerevisae*, CWI and plasma membrane damage initiate downstream responses, culminating in a G1/S cell cycle arrest, with receptors Mid2 and Wsc1 playing key roles in damage detection. Similarly, in humans such as *Homo sapiens*, damage to the extracellular matrix is detected by integrins, which trigger comparable transduction cascades resulting in G1/S cell cycle arrest, a process also elicited by extracellular matrix modifications via metalloproteinases (MMPs). The TGF-β pathway also control cell proliferation, regulating ECM synthesis and degradation, and modulating tissue remodeling processes. Integrins, as primary receptors for ECM proteins, establish bidirectional communication with growth factor and cytokine receptors. In plants like *Arabidopsis thaliana*, varied pathways respond to folding and wounding, influencing cell elongation or division and prompting cell wall remodeling, detected by THESEUS1 (THE1). FERONIA (FER) could also play a role due to its effect on auxin concentrations, however, while implied, the explicit link to the cell cycle checkpoint remains to be confirmed (dotted lines)

Understanding the relationship between the extracellular matrix and cell cycle regulation is essential since any perturbations on the first can have deep impacts on the latter. This section provides an overview of this interplay in organisms like yeast and mammals, serving as a comparative foundation for plants. We will delve into specific mechanisms such as yeast’s CWI system and its effects on cell cycle progression, mammalian integrins’ role in modulating cell cycle phases, and the influence of matrix metalloproteinases (MMPs) on extracellular signaling and cell cycle regulation. This knowledge provides a platform to compare these mechanisms in plant systems (Fig. 2).

### Cell wall/extracellular matrix control of cell cycle progression in the budding yeast

In the model yeast *Saccharomyces cerevisiae*, CWI signaling is intricately involved in various cellular processes. It influences processes such as cell growth, cytokinesis, and cell separation. Defects in CWI signaling can lead to impaired cell separation during the cell cycle, as observed in mutant strains lacking key components of the CWI pathway (González-Rubio et al. 2023).

Key CWI sensors like Mid2 and Wsc1 initiate the response to cell wall stress (Levin 2011). These sensors activate Rho1, a GTPase, through the guanine nucleotide exchange factor Rom2 (Bickle 1998). Rho1 then triggers a cascade involving several elements: activation of the protein kinase C (Pkc1), followed by the MAP kinase cascade components Bck1, Mkk1/2, and the MAP kinase Slt2 (Bickle 1998; Kono et al. 2012). This pathway culminates in the activation of transcription factors Swi4 and Swi6, which regulate the activity of G1-specific cyclin genes and ensure the entry into the mitotic cycle (Fig. 2) (Nasmyth and Dirick 1991; Kim et al. 2010). In addition to its role in cell wall remodeling, the CWI pathway significantly influences cell cycle progression (Quilis et al. 2021). The activation state of Mpk1 is closely linked to cell cycle regulatory proteins such as Cdc28, indicating a functional intersection between cell wall integrity and cell cycle regulation (Levin 2011). This pathway is intricately regulated throughout the cell cycle, with its signaling notably peaking during bud emergence, a critical phase for cell wall integrity and remodeling. Pkc1 not only responds to cell wall stress but also impacts nuclear functions, including the arrest of secretion response and G2/M progression, and phospholipid biosynthesis, further underscoring the pathway's expansive role in cell cycle progression and cellular integrity (Levin 2011). Protein phosphatases, such as Ptc1, negatively regulate MAPK pathways by dephosphorylating cascade components (González-Rubio et al. 2019). Ptc1 specifically dephosphorylates Mkk1 in the CWI pathway (Jiang et al. 1995; Du et al. 2006). The absence of Ptc1 leads to functional defects associated with CWI pathway activation, including altered growth, cell separation, and mitochondrial inheritance (Du et al. 2006; González et al. 2006; Li et al. 2010; Tatjer et al. 2016). Ptc1 also affects other physiological processes, such as the target of the rapamycin 1 (TORC1) complex, which regulates nutrient availability and cell proliferation (González et al. 2009). The mechanisms underlying these effects, including the involvement of Slt2 kinase activity, are still being investigated (Sánchez-Adriá et al. 2022).

The plasma membrane and the cell wall both pose significant challenges to maintaining cell integrity. Yeast CWI pathways are capable of sensing and responding to membrane damage (Bickle 1998). Plasma membrane damage activates a novel G1 checkpoint that involves the Mck1-dependent degradation of Cdc6 and stabilization of Sic1 (Al-Zain et al. 2015). Mck1, a yeast glycogen synthase kinase-3 (GSK-3) kinase, plays a crucial role in ensuring proper DNA replication, preventing DNA damage, and maintaining genome integrity by inhibiting Cdc6 (Fig. 2) (Ikui et al. 2012).

This intricate network, from CWI perception by sensors to the eventual cellular response mediated by transcription factors, underscores the importance of CWI in maintaining cell structure and function. Understanding these mechanisms in yeast provides valuable insights into similar processes in plants, enriching our grasp of how cells maintain integrity against environmental challenges.

### Extracellular matrix control of cell cycle progression in mammals

#### Integrins and focal adhesion kinase (FAK)

Integrins are adhesive receptors that play a critical role in cell cycle regulation by detecting and responding to signals from the extracellular matrix (ECM). They are essential for cell adhesion, survival, proliferation, differentiation, and migration (Moreno-Layseca and Streuli 2014). Integrins are heterodimeric receptors composed of one of 18 α and 8 β subunits. They are activated by ligand binding and mechanical force, which induces a conformational shift mediated by cytoplasmic proteins such as talin and kindlin (Lagarrigue et al. 2020, 2022; Lu et al. 2022).

Integrin engagement with the ECM leads to the formation of various adhesion complexes, including focal adhesions (FAs) and the activation of focal adhesion kinase (FAK). This activation, involving FAK’s trans-autophosphorylation at Tyr-397, initiates various downstream signaling pathways crucial for cell cycle regulation (Calalb et al. 1995; Acebrón et al. 2020). FAK, upon activation, phosphorylates targets like cyclin-dependent kinase inhibitors (CDKIs) p27 and p21, as well as cyclins D1 and A2, facilitating the transition from the G1 to the S phase of the cell cycle (Fig. 2) (Walker and Assoian 2005; Moreno-Layseca and Streuli 2014; Jones et al. 2019). During the G1 to S transition, integrin-FAK signaling activates the PI3K/AKT and MAPK/ERK pathways, upregulating cyclin D and degrading CDKIs to promote cell cycle progression (Zhu et al. 1996; Brunet 1999; Shanmugasundaram et al. 2013). Additionally, cyclin A2/CDK1 regulates FA and actin filament dynamics, crucial for FA growth, stability, and cell morphology changes necessary for mitosis entry (Jones et al. 2018; Gough et al. 2021). In the G2 phase, integrin adhesion influences the stimulation of PLK1, aiding in the transition to mitosis, with changes in cell morphology and traction forces being essential for successful cell division (Vianay et al. 2018; Kamranvar et al. 2022).

In plant cells, while integrins are not present, analogous mechanisms involving cell wall integrity receptors play a similar role in perceiving the extracellular environment. Plant CWI receptors detect changes in the cell wall composition and structure, triggering signaling pathways. These receptors, in a similar way to integrins in animal cells, sense mechanical signals from the cell wall, influencing cellular processes (Bacete and Hamann 2020).

#### Cytokines and growth factors

In animals, growth factors and cytokines in animals play a critical role in the regulation of the cell cycle, especially through their interactions with the ECM. The ECM serves as a major reservoir of these signaling molecules, which are bound within the matrix along with bioactive fragments produced from MMPs (Hynes 2009). This sequestration and subsequent release of growth factors from the ECM influence immune cell proliferation and differentiation, directly impacting cell cycle progression. Growth factors, binding to receptor tyrosine kinases (RTKs), initiate critical cell cycle events. These RTKs activate downstream signaling pathways, controlling CDK-cyclin complex activities, and influencing essential cellular functions such as migration, survival, and differentiation (Jones & Kazlauskas 2000; Wee & Wang 2017). Dysregulation in these pathways, as seen in notable RTK families like the EGF receptor, insulin receptor, PDGF receptor, and NGF receptor, often results in cancerous growth (Wang et al. 2017; Wee & Wang 2017).

Among the various ECM-bound molecules, transforming growth factor-beta (TGF-β) is particularly noteworthy for its multifaceted roles in development, tissue repair, and immune cell function. TGF-β interacts with its receptors to activate Smad proteins, which then regulate gene transcription, balancing ECM production and degradation. (Neuzillet et al. 2015; Meng et al. 2016; David and Massagué, 2018). The signaling cascade ultimately leads to the imbalance between ECM production and degradation, modulating tissue remodeling processes (Frangogiannis 2020).

In plants, while growth factors as such are not present, analogous signaling mechanisms involving the cell wall play a significant role in development. For example, Rapid Alkalinization Factor (RALF) peptides in plants function in a reminiscent manner to growth factors in animals (Blackburn et al. 2020). The recent insights into the LRX8-RALF4 complex in plants offer a striking example (Moussu et al. 2023). This complex’s interaction with demethylesterified pectins in the cell wall, leading to a reticulated network essential for cell wall integrity and expansion, mirrors the TGF-β pathway’s role in ECM modulation. Just as the TGF-β pathway influences the ECM and thus affects cell behavior in animals, the LRX8-RALF4-pectin interaction in plants is a critical determinant of cell wall structure, impacting cell growth and development, particularly in processes like pollen tube growth.

#### Matrix metalloproteinases (MMPs)

MMPs, a family of zinc-dependent endopeptidases, play a pivotal role in ECM remodeling, impacting a range of physiological processes from embryonic development to wound healing (Cabral-Pacheco et al. 2020; Chan et al. 2020; Laghezza et al. 2020). In mammals, MMPs, expressed in various tissues and cell types, not only contribute to cell cycle regulation by processing growth factors and signaling molecules (see section above), but also they remodel the ECM by degrading components such as collagens and fibronectin, influencing cell adhesion, migration, and growth factor availability, thereby impacting cell cycle progression (Lu et al. 2011; Kleiser and Nyström, 2020). Tissue inhibitors of metalloproteinases (TIMPs), a family of proteins, serve to bind and inhibit MMP activity. Maintaining the balance between MMPs and TIMPs is essential for ECM homeostasis (Baker et al. 2002; Cabral-Pacheco et al. 2020). MMPs also influence intracellular signaling pathways regulating the cell cycle by cleaving and modifying ECM-bound integrins (Fig. 2) (Kleiser and Nyström, 2020).

Some MMPs display cell cycle-associated expression patterns. For example, MMP-2 and MMP-9 participate in different cell cycle phases, with MMP-2 upregulated during the G1/S transition and MMP-9 during the G2/M transition, and disruptions in this equilibrium contribute to ECM degradation and alterations in cell cycle progression in different diseases (Vu and Werb 2000; Wang et al. 2017; Cabral-Pacheco et al. 2020).

In plants, enzymes like cellulases, pectinases, and expansins modulate the physical properties of the cell wall (Cosgrove 2022), akin to how MMPs modulate ECM composition in animals. This remodeling is crucial for facilitating cell growth and expansion, although it is yet to be explored how or if these control cell cycle transitions.

## Current understanding of cell wall integrity and cell cycle regulation in plants

The exploration of the connection between plant CWI and cell cycle regulation is a rapidly evolving area of research. Drawing parallels from the established knowledge in other organisms, it is becoming increasingly evident that various signaling molecules and extracellular modifications are intricately linked with complex molecular mechanisms in plants. Furthermore, the entwined hormonal networks, fundamental to both cell cycle progression and cell wall biosynthesis and remodeling, present potential avenues for understanding this interplay. By delving into these connections, we can gain profound insights into the significance of CWI in orchestrating cell cycle progression and influencing plant growth. In this section, we collate and examine the growing body of evidence that underscores this intricate relationship, highlighting the intricate dance of cellular processes that govern plant development and adaptation.

### Cytokinins, CYCD3;1, and NIA1/NIA2

Cytokinins are key plant hormones that play a critical role in various aspects of plant growth and development. Their primary function is promoting cell division, particularly in plant roots and shoots, but their influence extends far beyond this process (Mok and Mok 2001). Cytokinins regulate leaf senescence, apical dominance, nutrient assimilation, and response to environmental stresses (Werner et al. 2001; Rivero et al. 2007).

Cytokinins are fundamental in regulating the plant cell cycle, particularly in controlling key phase transitions in response to environmental stresses such as drought (Skirycz et al. 2011; Tenhaken 2015). During the G1 to S phase transition, cytokinins play a significant role by modulating the expression of D-type cyclins, such as CYCD3;1. CYCD3;1 is essential for the initiation of DNA replication and is a key regulator of the G1/S checkpoint in the plant cell cycle (Fig. 2) (Riou-Khamlichi et al. 1999). In the G2 to M phase transition, cytokinins modulate the activity of CDKs and their associated cyclins, which are crucial for mitotic entry. Cytokinins can also influence the levels of specific B-type cyclins, which are essential for the G2 to M transition (Boruc et al. 2010). Furthermore, cytokinins have been implicated in the regulation of the retinoblastoma-related (RBR). This pathway is a critical regulator of the G1/S transition, with RBR proteins interacting with D-type cyclins like CYCD3;1 (Boruc et al. 2010).

In addition to their direct impact on cell cycle regulators, cytokinins also interact with other hormonal pathways, such as those mediated by auxins, to fine-tune cell cycle progression (Perilli et al. 2010). This interaction exemplifies the complex network of signaling pathways that converge to regulate the plant cell cycle, with cytokinins playing a central role. Another example of the interaction of cytokinins with other signaling molecules is nitric oxide (NO). Cytokinins and NO can have both synergistic and antagonistic effects on plant growth and development (Freschi 2013; Shen et al. 2013). For instance, during cell division regulation, NO participates in callus formation and shoot regeneration by activating CYCD3;1 at the G1-S cell-cycle phase transition. On the other hand, NO antagonistically affects root growth, as overproduction of NO inhibits root development (Shen et al. 2013) A role for NO in cytokinin signaling has also been suggested for controlling plant cell death (PCD), possibly through the inhibition of mitochondrial respiration (Carimi et al. 2005). Furthermore, NO-overproducing Arabidopsis lines and mutant plants show reduced sensitivity to cytokinins, leading to a negative regulation of cytokinin signaling through S-nitrosylation of phosphotransferprotein1 (AHP1), thereby repressing phosphorylation activity during cytokinin-mediated phosphorelay (Feng et al. 2013).

Nitrate reductase 1 (NIA1) and NIA2 genes, are key players in this regulatory network. These enzymes are responsible for the reduction of nitrate (NO3-) to nitrite (NO2-), and subsequently to NO (Wilkinson and Crawford 1993). Cytokinin treatment in plants has been shown to increase NO levels, which is thought to be mediated by the activation of nitrate reductase enzymes encoded by NIA1 and NIA2 (Yu et al. 1998; Tun et al. 2001). Furthermore, the interaction is bidirectional. NO, possibly produced via NIA1 and NIA2 activity, can influence cytokinin signaling. In tobacco leaves, the application of NO donors affects the expression of cytokinin-responsive genes, indicating that NO signaling can modulate cytokinin response pathways (Tun et al. 2001). This modulation by NO is also evident in processes like root growth, where high levels of NO can antagonize cytokinin signaling, thereby affecting root development (Fernández-Marcos et al. 2011).

Interestingly, recent studies have provided some initial evidence supporting the connection between CWI, cytokinins, NO and cell cycle activity in plants. For instance, Arabidopsis NIA1/NIA2, CYCD3;1, and cytokinins have been implicated in this coordination (Gigli-Bisceglia et al. 2018). In a study investigating the impact of cell wall damage on *A. thaliana* seedlings, researchers found that cell wall damage inhibited cell cycle gene expression and increased transition zone cell width in an osmosensitive manner (Gigli-Bisceglia et al. 2018). These results were correlated with cell wall damage-induced changes in cytokinin homeostasis, specifically the upregulation of CYTOKININ OXIDASE/DEHYDROGENASE 2 and 3 (CKX2, CKX3) transcript levels. Further investigations *using nitrate reductase1 nitrate reductase2* (*nia1 nia2*) seedlings revealed that the upregulation of CKX2 and CKX3 and the repression of cell cycle gene expression by cell wall damage were absent in these mutants, highlighting the role of NIA1/2-mediated processes in regulating cell wall damage responses (Fig. 2) (Gigli-Bisceglia et al. 2018). This study suggests that cell wall damage enhances cytokinin degradation rates through a NIA1/2-mediated process, leading to the attenuation of cell cycle gene expression.

### Auxin and restorative divisions after wounding in roots

Auxins play a pivotal role in regulating various aspects of plant growth and development. As one of the most important phytohormones, auxins are crucial in processes such as cell division, cell elongation, cell wall loosening and differentiation, influencing the overall plant morphology and adaptive growth responses (Tanimoto 2005; Majda and Robert 2018). In the context of cell cycle regulation, auxins exert a significant influence by controlling the transition of cells from the G1 phase to the S phase by regulating the expression of various cell cycle genes, including those encoding for D-type cyclins and CDKs (Fig. 2) (Perrot-Rechenmann 2010). Auxins also interact with other signaling pathways and hormones, such as cytokinins and gibberellins, to finely tune the cell cycle and ensure coordinated growth and development (Mazzoni-Putman et al. 2021). One of the most intriguing aspects of auxin biology is its role in spatial patterning within plant tissues. Auxin gradients are established through its polar transport, leading to differential growth responses in different parts of the plant (Galvan-Ampudia et al. 2020). This directional movement of auxin is fundamental in shaping plant architecture, including the formation of leaves, flowers, and roots.

Plant cells, unable to migrate, rely on targeted cell division and expansion for wound regeneration. Wound healing in plant tissues involves unique mechanisms distinct from those in animals that encompass the detection of the damage (a process related to CWI monitoring) and the coordination of the restorative divisions (Hoermayer et al. 2020). Auxin signaling has been implicated in restorative divisions following wounding in roots. Live imaging studies using laser-based wounding in Arabidopsis’s root provided mechanistic insights into wound perception and coordination of wound responses. The collapse of damaged cells contributes significantly to wound perception, and a specific increase in auxin levels was detected in cells adjacent to the wound. This localized auxin increase plays a dose-dependent role in balancing wound-induced cell expansion and restorative division rates, preventing tumorous overproliferation (Hoermayer et al. 2020). Disruption of the canonical TIR1 auxin signaling pathway leads to dysregulation of these processes. Furthermore, auxin and wound-induced turgor pressure changes spatially define the activation of key components of regeneration, such as the transcription regulator ERF115 (Fig. 2) (Hoermayer et al. 2020). Mechanical cues have been shown to influence ERF114 and ERF115 expression, which correlates with BZR1-mediated brassinosteroid signaling under both regenerative and developmental conditions. Interestingly, CWI surveillance via the Catharanthus roseus receptor-like kinase 1-like (CrRLK1L) CWI sensor FERONIA (FER) antagonistically suppresses their expression in both scenarios, suggesting a molecular framework where cell wall signals and mechanical strains regulate organ development and regenerative responses through ERF114- and ERF115-mediated auxin signaling (Canher et al. 2022). These findings suggest that CWI and wound signaling involves the sensing of damaged cell collapse and the activation of local auxin signaling to coordinate downstream transcriptional responses in the immediate vicinity of the wound.

### Endoreplication and cell wall composition

The relationship between endoreplication and cell wall composition has recently attracted attention (Bhosale et al. 2019). It has been observed that ploidy levels often scale with the final size of cells and organs, suggesting the involvement of endoreplication in these processes (Orr-Weaver 2015). However, exceptions to this correlation exist, and the exact nexus between endoreplication and size regulation remains elusive.

Previous studies have revealed that endoreplication plays a significant role in apical hook folding in Arabidopsis. This process is influenced by variations in growth, primarily caused by differences in the distribution of the phytohormone auxin and the mechanical properties of the cell wall. Specifically, the inner cells, which contain high auxin concentrations and stiffer walls, experience suppressed elongation. On the other hand, the rapidly growing outer cells exhibit lower auxin levels and softer walls, allowing for their continued expansion (Baral et al. 2021; Jonsson et al. 2021). Furthermore, a molecular pathway has been identified, linking endoreplication levels to cell size through cell wall remodeling and stiffness modulation. Remarkably, endoreplication is not solely permissive for growth; reducing endoreplication levels enhances wall stiffening and actively reduces cell size. The feedback loop involved in this process is mediated by the CrRLK1L CWI receptor THESEUS1 (THE1) (Ma et al. 2022). These findings provide insights into the nonlinear relationship between ploidy levels and size and offer a molecular mechanism that connects mechanochemical signaling with endoreplication-mediated dynamic control of cell growth.

Although the contribution of ploidy levels to cell growth has been debated, accumulating evidence suggests that the onset of the endocycle, the initiation of endoreplication, may influence cell growth through the transcriptional control of cell wall-modifying genes (Bhosale et al. 2019). This transcriptional regulation is believed to drive changes in the cell wall structure, allowing for the expansion required to accommodate turgor-driven rapid cell expansion. It supports the notion that vacuolar expansion, rather than a ploidy-dependent increase in cellular volume, represents the primary force driving cell growth (Bhosale et al. 2019). Understanding the interplay between endoreplication, transcriptional control of cell wall-modifying genes, and vacuolar expansion provides valuable insights into the mechanisms underlying cell growth in plants. It highlights the importance of cell wall composition and dynamics in maintaining CWI and facilitating cell expansion.

### Recent findings on molecular mechanisms linking CWI perception, cell wall biosynthesis, and gene expression regulation

**Fig. 3:**
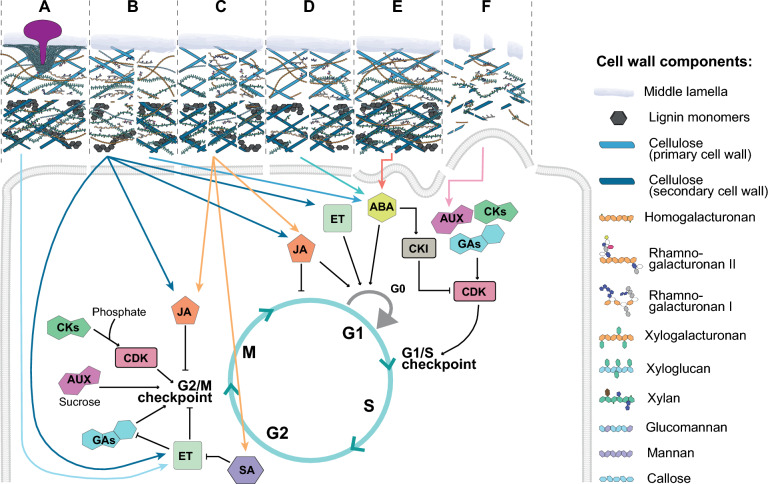
Alterations in different cell wall components could be integrated with cell cycle progression checkpoints. Cell wall mutants with alterations in biosynthesis or deposition of callose (A), cellulose (B), pectins (C), hemicelluloses (D), as well as seedlings under hyperosmotic stress (E) or wounded (F) activate different hormonal pathways which in turn control cell cycle progression. *CK* cytokinins, *AUX* auxins, *GAs* gibberellins, *JA* jasmonic acid, *ET* ethylene, *SA* salicylic acid, *ABA* abscisic acid, *CDK* cyclin-dependent kinase, *CKI* cyclin-dependent kinase inhibitor, *CycD* D-type cyclin

Plant cell walls play a vital role in maintaining plant structure, safeguarding against various stresses, and facilitating cell-to-cell communication. The CWI monitoring system is essential for sensing mechanochemical changes in the cell wall. It triggers signaling pathways in response, establishing a feedback loop between the living cell’s protoplast and the extracellular matrix of the cell wall, known as the apoplast (Fig. 3). The current body of evidence suggests that CWI perturbations can be detected by the CWI monitoring system through the perception of cell wall fragments by receptor like kinases (RLKs) and receptor like proteins (RLPs), distortion of the cell wall-plasma membrane continuum, or displacement of the plasma membrane versus the cell wall (Bacete and Hamann 2020).

To date, only the RLK THE1 has been directly associated with both CWI monitoring and a cell-cycle-related process as endoreplication (Ma et al. 2022). Interestingly, THE1 has emerged as a potential CWI mechanoreceptor, playing a crucial role in coordinating cell wall mechanics and processes, such as the regulation of abscisic acid production, a hormone vital for plant stress response and developmental cues (Bacete et al. 2022). The intriguing aspect of THE1’s function is its potential to integrate both chemical and physical signals, mirroring how integrins in animals serve as connectors between the internal cellular environment and external matrix. However, this does not exclude a potential role of other CWI RLKs/RLPs in the coordination of CWI and cell cycle progression. In particular, FER seems a promising candidate for this, giving its role in mechanosensitive auxin signaling (Canher et al. 2022).

In the intricate interplay between plant development and environmental adaptation, the plant cell wall stands out not just as a structural barrier but as a dynamic mediator in cellular processes, including hormonal pathways (Jonsson et al. 2022). This offers a potential link to how cell wall perturbations can influence the cell cycle through hormonal pathways.

The response of the cell wall to environmental stimuli, such as pathogen attacks, exemplifies this dynamic relationship. For instance, when faced with pathogen stress, plants reinforce their cell walls by depositing callose. This adaptive mechanism not only strengthens the cell wall but also intricately triggers ethylene (ET) pathways, essential for plant immunity (Voigt 2014). This phenomenon is part of a broader context where modifications in the cell wall components, like cellulose and hemicelluloses have profound effects on the balance and signaling of hormones such as ET, jasmonic acid (JA), salicylic acid (SA), and abscisic acid (ABA) (Fig. 3) (Bacete et al. 2018). Pectin modifications in the cell wall also trigger hormonal responses involving JA and SA, which are crucial for the plant’s adaptation to environmental changes. The role of wall-associated kinases (WAKs) in sensing these modifications, leading to the production of SA and/or JA, links the mechanical state of the cell wall to biochemical signaling pathways (Kohorn 2016).

The influence of these hormonal changes extends to critical phases of the cell cycle. Hormones like JA, cytokinins, auxins, gibberellins (GAs), and ET are particularly influential at the G2/M checkpoint (Fig. 3), a key phase in regulating the transition from growth to cell division (Shimotohno et al. 2021). The intricate hormonal interplay, such as the cross-effects effect of SA on ET and JA pathways and the impact of JA on the switch between mitotic cycle and endocycle (Patil et al. 2014), highlights the complex regulatory mechanisms plants employ. Moreover, JA, along with ET and ABA, influences the entry in G0 phase (Velappan et al. 2017). Conversely, auxins, cytokinins, and GAs are key regulators of CDKs and play a vital role in the G1/S checkpoint (Fig. 3) (Shimotohno et al. 2021). ABA’s role extends to promoting the expression of CKI-coding genes such as ICK1/KPR1, pivotal in cell cycle control (Wang et al. 1998). The cell-wall-dependent production of ABA in response to hyperosmotic stress exemplifies the cell wall’s role in perceiving environmental changes and triggering appropriate hormonal responses that could influence cell cycle progression (Bacete et al. 2022). Conversely, the role of these hormones in modifying the cell wall, impacting properties like extensibility and strength, is integral for plant growth and development. This dynamic nature of the cell wall, in conjunction with hormonal signaling, underscores its critical role in not just supporting plant structure but actively participating in the regulation of the cell cycle and adaptation to environmental challenges.

Transcriptional regulation of cell wall metabolism is closely linked to CWI signaling and involves several key components. It has been primarily observed in the context of immune responses. For instance, FER, upon interaction with its ligands (RALF peptides) phosphorylate the transcription factor MYC2, influencing JA signaling (Guo et al. 2018). Looking at transcription factors as an end point of CWI-related pathways could be an interesting perspective to look for candidates for CWI-cell cycle coordination. Notably, the transcription factor MYB46 in *A. thaliana* has emerged as a key player, orchestrating cell growth and cell cycle progression. MYB46 expression is induced upon wounding, leading to the upregulation of genes associated with cell wall biosynthesis and the cell cycle (Shi et al. 2021). This coordinated response promotes the biosynthesis of the cell wall by enhancing the expression of cell wall-associated genes. Also, it upregulates a battery of genes involved in cell cycle progression (Shi et al. 2021). The involvement of MYB46 in this regulatory network has been observed in seven plant species harboring R2R3-MYB domains, including *A. thaliana*, *Fragaria vesca* (strawberry), *Malus domestica* (apple), *Prunus mume* (plum blossom), *Prunus persica* (peach), *Pyrus bretschneideri* (pear), and *Rosa chinensis* (China rose), highlighting its evolutionary conservation (Shi et al. 2021).

## Future perspectives and unanswered questions

As we have discussed above, CWI is a complex process that involves the perception of physical and chemical stimuli. Disturbances in cell wall homeostasis are CWI receptors, initiating various signal transduction pathways that allow plants to identify the origin of such disturbances—either environmental or developmental—and respond appropriately (Bacete and Hamann 2020). However, our understanding of CWI and its monitoring system remains partial, derived from diverse studies across various tissues, organs, and species (Vaahtera et al. 2019), hindering our comprehensive knowledge of the mechanisms involved. In the context of their influence on cell cycle progression, an interesting approach could look at homologs of the yeast and animal signaling cascades described above and summarized in Fig. 2. After all, this approach has been successfully exploited in the past to characterize CWI mechanisms in plants (Hamann and Denness 2011).

Furthermore, compensatory modifications to the cell walls often serve as a common response to CWI disturbances, as the plant tries to restore cell wall functionality (Denness et al. 2011). These modifications can act as both the trigger for the CWI monitoring system (input) or the result of this system’s activation (output). The distinction lies solely in the temporal dimension, emphasizing the importance of considering this aspect for a comprehensive understanding of CWI. Yet, the technical challenges associated with studying dynamic processes remain substantial, due to the lack of spatial and temporal resolution of the employed methods (Alonso Baez and Bacete 2023). A nice example of how high-resolution techniques can shed light into how cell wall properties relate to cell division and morphogenesis is the recent study by (Bonfanti et al. 2023). By employing time-lapse imaging and atomic force microscopy, the authors systematically mapped the stiffness of cell walls in relation to their age and growth in *Marchantia polymorpha and A. thaliana*. Intriguingly, it was found that new walls in *M. polymorpha* gemmae become transiently stiffer and slower-growing compared to older walls, a phenomenon not observed in Arabidopsis leaves. This differential behaviour impacts local cell geometry and junction angles, underlining the significance of cell wall mechanics in plant morphogenesis. Further studies in this direction and with this level of resolution can provide interesting insights into how CWI and cell cycle progression impact each other.

The “growth-defense trade-off” in plant biology underscores the strategic allocation of resources, intricately governed by a network of phytohormones and cell division control. Essentially, plants must decide whether to allocate resources towards growth or defense against various stresses. This trade-off is particularly evident in the context of CWI maintenance. For instance, certain changes in cell wall composition not only enhance stress resistance but can also boost biomass and seed production. The Arabidopsis mutant *arr6* serves as a compelling example, displaying modified CWI responses alongside an optimized growth-defense balance (Bacete et al. 2020). However, the exact mechanisms driving this trade-off are not fully understood, and manipulating the cell wall doesn’t always enhance plant growth. There is no commonly accepted underlying mechanism for these growth defects, and new approaches are needed to better understand how changes in cell wall composition may quantitatively affect growth. The perspective of cell cycle and cell division playing a significant role in this interaction is a compelling idea and might or might not involve hormonal balance. This knowledge could revolutionize our approach to agriculture, improving crop performance in the face of an increasingly changing environment, and thereby contributing to sustainable food security.

## Conclusion

In this review, we provide an extensive overview of the current understanding of the critical relationship between CWI and cell cycle progression in plants. The mechanisms underlying this relationship are multifaceted and complex, involving a wide array of genes, transcription factors, and signaling pathways, underpinning everything from cell wall biosynthesis to CWI perception and adaptive responses.

The plant cell wall, far from being an inert, passive barrier, has emerged as a dynamic and responsive structure that intimately links the physical state of the cell with a myriad of developmental and stress response processes. The complex interplay between CWI maintenance, the cell cycle, and gene expression regulation is central to plant growth and development, with the potential to influence plant resistance to environmental stressors. However, much remains to be elucidated. Key questions persist around the specific CWI sensing mechanisms in plants and the role of CWI maintenance in the growth-defense trade-off. Likewise, our understanding of how these processes might be conserved or divergent across species is still in its infancy. Continued research into these areas will not only shed light on fundamental biological processes but also have the potential to generate practical applications for crop improvement and sustainable agriculture. Ultimately, the potential implications of this research are profound. By connecting our growing understanding of CWI to cell cycle coordination, we could enhance plant productivity and resilience, providing more robust responses to a rapidly changing environment. This would have significant implications for global food security and could contribute to more sustainable agricultural practices.

In conclusion, although we have made significant strides in understanding the intricate dance between CWI and cell cycle progression in plants, this field remains ripe for exploration. As we venture forward, each new discovery not only reveals more about the complex biology of plants but also brings us a step closer to harnessing these insights for the betterment of agriculture and, ultimately, society.

## Data Availability

Not applicable.
